# Grape RNA-Seq analysis pipeline environment

**DOI:** 10.1093/bioinformatics/btt016

**Published:** 2013-01-17

**Authors:** David G. Knowles, Maik Röder, Angelika Merkel, Roderic Guigó

**Affiliations:** ^1^Bioinformatics and Genomics Group, Centre for Genomic Regulation (CRG) and ^2^Universitat Pompeu Fabra (UPF), Dr Aiguader 88, 08003 Barcelona, Spain

## Abstract

**Motivation:** The avalanche of data arriving since the development of NGS technologies have prompted the need for developing fast, accurate and easily automated bioinformatic tools capable of dealing with massive datasets. Among the most productive applications of NGS technologies is the sequencing of cellular RNA, known as RNA-Seq. Although RNA-Seq provides similar or superior dynamic range than microarrays at similar or lower cost, the lack of standard and user-friendly pipelines is a bottleneck preventing RNA-Seq from becoming the standard for transcriptome analysis.

**Results:** In this work we present a pipeline for processing and analyzing RNA-Seq data, that we have named Grape (Grape RNA-Seq Analysis Pipeline Environment). Grape supports raw sequencing reads produced by a variety of technologies, either in FASTA or FASTQ format, or as prealigned reads in SAM/BAM format. A minimal Grape configuration consists of the file location of the raw sequencing reads, the genome of the species and the corresponding gene and transcript annotation.

Grape first runs a set of quality control steps, and then aligns the reads to the genome, a step that is omitted for prealigned read formats. Grape next estimates gene and transcript expression levels, calculates exon inclusion levels and identifies novel transcripts.

Grape can be run on a single computer or in parallel on a computer cluster. It is distributed with specific mapping and quantification tools, but given its modular design, any tool supporting popular data interchange formats can be integrated.

**Availability:** Grape can be obtained from the Bioinformatics and Genomics website at: http://big.crg.cat/services/grape.

**Contact:** david.gonzalez@crg.eu or roderic.guigo@crg.eu

## 1 INTRODUCTION

The development of ultrasequencing technologies during the recent years has started a major revolution in Biology. The ability to directly survey the cell’s RNA content by applying NGS technologies to cDNA sequencing (‘RNA-Seq’) has provided insights of unprecedented depth on the transcription landscape of many species (Wang *et al.*, 2009) such as *Homo sapiens* (Wang *et al.*, 2008; Sultan *et al.*, 2008; Montgomery *et al.*, 2010; Trapnell *et al.*, 2010), *Mus musculus* (Mortazavi *et al.*, 2008; Guttman *et al.*, 2010), *Arabidopsis thaliana* (Lister *et al.*, 2008), *Saccharomyces cerevisiae* (Nagalakshmi *et al.*, 2008; Yassour *et al.*, 2009) and *Schizosaccharomyces pombe* (Castle *et al.*, 2008). RNA-Seq has proven particularly powerful on tasks such as identifying novel genes and novel splice forms, detecting low abundance transcripts and finding sequence variations, such as SNPs (Marguerat *et al.*, 2008). It is gradually substituting microarrays as the technology of choice for transcriptome analyses, providing access to a greater dynamic range of RNA expression levels (Marioni *et al.*, 2008).

The throughput of NGS technologies is continuously accelerating, and the cost per sequenced nucleotide is rapidly falling. As a consequence, an unprecedented amount of data are being produced, pressing for the development of fast and efficient methods of analysis, as the bottleneck for scientific discovery is gradually shifting from data production to data analysis.

In the analysis of NGS data, processing efficiency is governed predominantly by two factors: first, the sheer size of the individual datasets, and second, the vast number of datasets to be analyzed. The current generation of sequencing machines, for example, Illumina HighSeq, can produce the equivalent of 20*×* the coverage of the human genome in a single run, delivering ∼600 million reads with a length of >100 nt. This has spawned a new generation of aligners optimized for aligning short sequences to the genome, for example, Bowtie (Langmead *et al.*, 2009), BWA (Li and Durbin, 2009), BFAST (Homer *et al.*, 2009) and GEM (Marco-Sola *et al.*, 2012), which are much faster than previous tools such as Basic Local Alignment Search Tool, FASTA or SSEARCH (see Trapnell and Salzberg, 2009, for a short review). Additionally, an increasing number of projects involve large sample sizes to be analyzed, often under complex experimental designs.

In the specific case of RNA-Seq, mapping of reads is only the first step of a complex data processing schema, the final goal of which is to produce accurate gene and transcript quantifications, and to delineate novel transcript structures. The lack of easy-to-use pipelines to perform such a processing out of the box in a transparent and streamlined fashion is actually a bottleneck that prevents the expansion of RNA-Seq, and prompts users with little access to sophisticated bioinformatic resources to prefer microarrays, for which standard ready-to-use processing pipelines and user-friendly bioinformatic analysis tools exist.

To address this need, specific pipelines have recently been developed for analyzing RNA-Seq data, such as the pipeline developed by Goncalves *et al.* (Goncalves *et al.*, 2011), an analysis pipeline developed in R within the context of the ArrayExpress Database. These and other tools facilitate the analysis of RNA-Seq data, but still require the user to have a significant bioinformatics background, specifically when complex experiments with a large number of datasets need to be analyzed.

Here we describe Grape, a workflow for the analysis of RNA-Seq data that automates all the steps from RNA-Seq reads to transcript quantification and discovery. Its user-friendly interface provides the necessary overview of all the data, and is therefore particularly suited for processing numerous files, produced in complex experimental setups. The results, both intermediate and final, are stored in an MySQL database, allowing for access through the database interface, but can also be visualized through Raisin, a user-friendly web application. The same html interface that is used for standalone local analysis is easily deployed on a web server for remote access in collaborative projects, or data dissemination.

Grape takes three types of input files. First, the read files, which may be aligned or not, the reference genome sequence file and the corresponding gene annotation file. Alternatively, Grape can also take read alignment files as input, rather than raw read files. Grape produces a number of output files in tabular format, so they can be easily loaded into most statistical packages. While this is the case for quantifications, which are made available individually at the gene, transcript and exon level, the BAM format is used as an output format for alignments.

More specifically, processing of RNA-Seq reads by Grape involves the following steps: (i) sequence read evaluation, and trimming if required; (ii) mapping to the genome, and the transcriptome; (iii) assembly of reads in absence of a reference genome, or before genome mapping; (iv) identification of novel exons, and splice junctions and modeling of transcript structures and (v) gene and transcript quantification. Grape pays special attention to quality controls (QCs), which trace quality scores and uncalled bases along the reads, look for biased nucleotide composition and inspect the distribution of reads along transcripts. These can be used for QC, although Grape leaves any decision to modify the data, such as trimming and filtering, to the user.

Grape is based on the PIP pipeline management system (Conery *et al.*, 2005). The current Grape distribution uses SAMtools (Li *et al.*, 2009), the GEM mapper (Marco-Sola *et al.*, 2012), the Flux Capacitor (Montgomery *et al.*, 2010) and Cufflinks (Trapnell *et al.*, 2010), but any tool compliant with popular data interchange formats like GFF, BAM/SAM and BED can be used. Grape can be run locally or on a computer cluster. Speed-up of the analyses is achieved by parallelizing certain steps and taking advantage of multithreading where possible. The Grape implementation conserves a copy of the exact software and configuration used for a given set of analyses, guaranteeing forward reproducibility.

## 2 APPROACH

### 2.1 Grape description

Grape is an automated workflow integrating the management, analysis and visualization of RNA-Seq data. GRAPE can map the reads to the genome and/or transcriptome, and it can also work with single or paired end reads, both stranded or not.

It organizes the RNA-Seq datasets in projects, that is, sets of datasets, that are generated within the same study and are all analyzed using the same annotation files and reference genome sequence.

Two MySQL databases store the information generated by the individual steps in the pipeline, allowing for efficient storage and retrieval of data. One of these is used to store the metadata (cell type, RNA extract, etc) as well as information that is independent of the reads (junction libraries, indices location, etc), and the other for the results produced for each of the datasets.

Grape uses the Buildout package (http://www.buildout.org) to set up all necessary components for running the analysis, and Raisin to visualize the data through a web browser.

Buildout is a widely used Python package for assembling and deploying applications from multiple parts. In our case, Buildout installs all the programs used by Grape, like GEM and the Flux Capacitor, and makes the Grape pipeline scripts available to the individually configured RNA-Seq analysis pipelines. Each pipeline is linked to one of the datasets in the project, and has its own independent directory structure. The pipelines are run in parallel, and can be inspected through log files tracing all execution steps.

The only requirement for the Buildout is a set of configuration files containing information on the read files and parameters to be used during the analysis, such as annotation, number of mismatches allowed during mapping, location of data files, etc. These configuration files also serve as a way to track all analysis performed for a project and the metadata associated to a project. Most importantly, they allow future reproducibility of the results. Internally, we manage all project configurations within the version control system Subversion (SVN), so they can be easily updated and traced.

Buildout installs two central scripts that are needed for running the individual pipelines. The first is the start.sh, which inserts the pipeline information into the metadata MySQL database. The second is the execute.sh script that runs the actual pipeline and stores all its results in the results MySQL database. The analyses are executed as so-called ‘rules’ through a template file (similar to a Makefile). Grape can be run on a single dataset or simultaneously on several datasets. The first *common* database holds all metadata concerning the pipeline runs, such as information regarding the input data. It is also used as a persistent cache for some shared data, like the library of splice junction coordinates retrieved from the annotation. The second database contains all information from the individual runs that are specific to each dataset, and includes the statistics needed for subsequent analysis. Finally, it stores the time stamp of each executed rule of a dataset, which makes Grape capable of resuming work seamlessly after interruption.

Using a rule-based approach offers the flexibility of being able to select only a subset of steps to execute, while automatically devoting resources only to the inferred analysis steps. Each step contains a set of prerequisite steps that need to be completed before the step itself can be executed, and a set of instructions that will be performed by the step. This information, encoded in a template file, is read by the execution script. This script builds a graph of dependencies between each of the different steps and executes those necessary to reach the required step. This allows Grape to perform all analysis steps in an ordered and easily reproducible manner, and at the same time, to store results and keep track of successfully completed steps This is particularly important when the aim is to analyze many large datasets in a consistent manner. An additional advantage is that the template file contains all the steps executed by the pipeline in a human readable way. By editing the template file, steps can be removed, added or modified.

Grape results include expression levels provided as reads per kilobase per million and/or unique read counts for different features, such as genes, transcripts, exons and junctions. They can be accessed through the command line by directly querying the Grape databases or through the Raisin web-application. Raisin is also based on Buildout, and uses the same configuration files used for producing the pipelines. Raisin accesses the results MySQL database created by Grape, and displays and summarizes information produced at the major steps in the analysis pipeline (see later in the text).

The Raisin web server comes with two configurations, one for accessing Grape locally on a workstation, and another, for dissemination of the results on a local Intranet or the Internet for collaborative projects.

Currently, RNA-Seq data processed by Grape from several public projects, such as the Illumina Body Map Project (HBM) or ENCODE (ENCODE Consortium 2004, 2011) can be browsed at http://rnaseq.crg.es (http://rnaseq.crg.es/project/HBM/tab/experiments and http://rnaseq.crg.es/project/ENCODE/tab/experiments/).

### 2.2 Analysis implemented in Grape

Here we describe the analysis steps implemented in Grape and the tools used to perform them. These tools are included in the standard distribution of Grape. However, alternative tools can be used for the analysis, provided they comply with popular input/output formats. To illustrate the Grape analyses, we use an RNA-Seq dataset from the Illumina Human Body Map (HBM) project (Additional information on the sample and the raw data can be obtained from http://www.ncbi.nlm.nih.gov/), with http://rnaseq.crg.es/project/ENCODE/tab/experiments/ as an example. Here, PolyA+/random primed RNA from a mixture of tissues was interrogated. Three different isolation methods were used: PolyA+, PolyA+ normalizing the RNA to more efficiently sample lowly expressed transcripts and Ribo minus instead of PolyA+ to remove the ribosomal RNA. The three samples were sequenced using the Illumina HiSeq 2000 at a read length of 100 nt. Each of these sequencing experiments produced, on average, 400 million reads. The Raisin interface to the output produced by the Grape analysis of this data is available at http://rnaseq.crg.es/project/HBM/tab/experiments/.

**Fig. 1.**
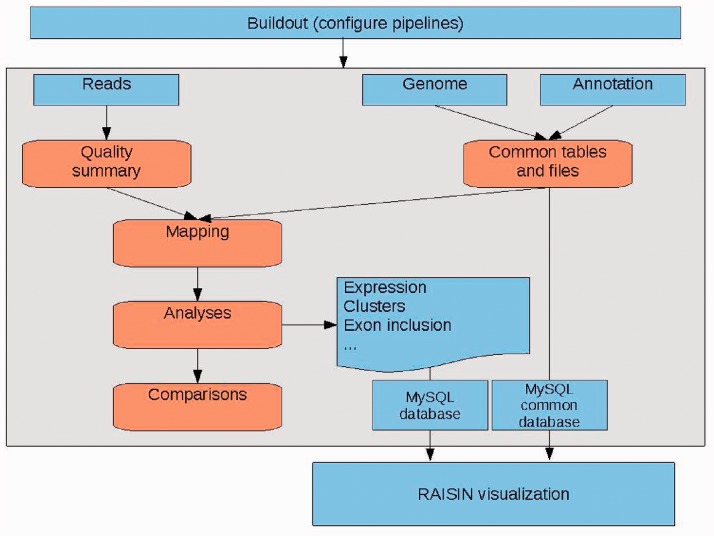
Overview of the three main components of the Grape RNA-Seq analysis pipeline environment. Buildout: performs the initial configuration. MySQL: two databases are used, one containing information specific to the datasets analyzed such as quantifications-detected elements, etc. Another contains information dependent on genome and annotation, as well as meta-information that allows for the linking of the different datasets. Raisin web application: allows the visualization of the analysis summaries using a web browser

The main steps in the Grape analysis (illustrated in Fig. 1) are as follows:

- Preprocessing and quality checks
- Mapping
- Post-mapping
- Transcript quantification
- Discovery and delineation of novel transcribed elements
- Summary statistics

**Preprocessing and quality checks:** Before processing the reads, Grape creates a number of files and database tables required for performing the analyses and storing the results. Experiments are organized according to the metadata given in the Buildout configuration files.

**Fig. 2.**
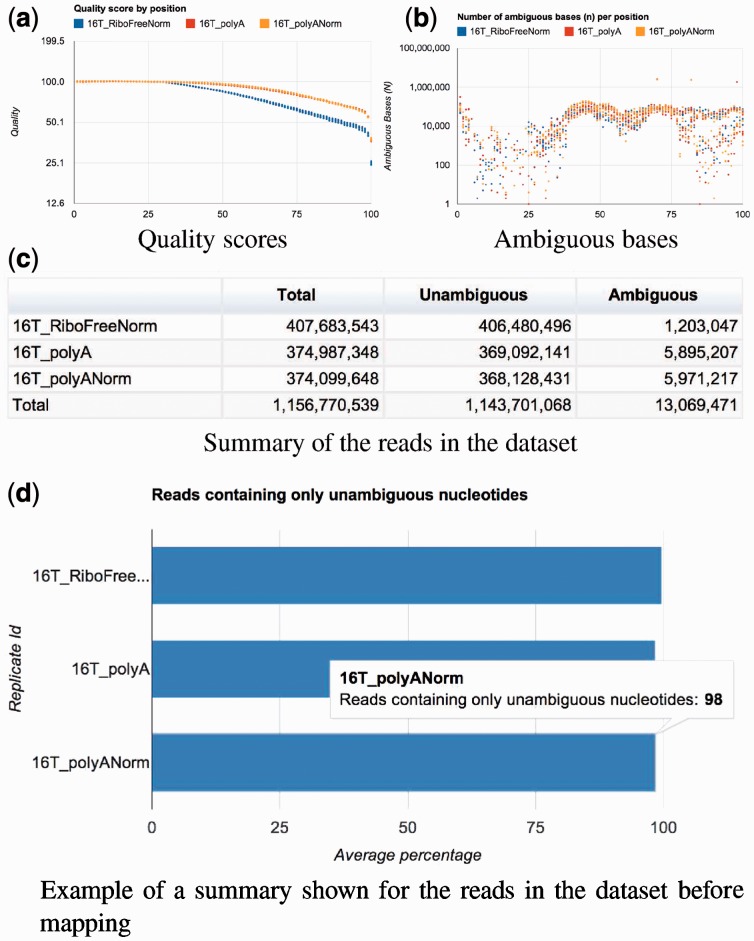
Raisin visualization of Grape’s QC step. Panels **a** and **b** show the distribution of quality scores and ambiguous nucleotides along the length of the reads. Panel **c** and **d** show summaries of the number of reads in the dataset as well as the fraction of reads with no ambiguous bases and the number of unique sequences as a percentage of the total

Next, Grape produces some basic statistics, and checks the quality of the RNA-Seq data by verifying the data format, calculating the distribution of ambiguous bases (bases where the sequencer was unable to call the base correctly and assigned it an N) and tracing quality scores along the reads. Figures 2a, b and d illustrate the Raisin interface to some of these QC steps. These initial QC steps contribute to assess whether additional preprocessing, such as trimming and/or filtering of the reads, is necessary. However, Grape only provides the QC information, and it is up to the user to decide whether additional preprocessing is required. Grape’s architecture allows for conveniently running it in stages. For example, Grape may be run first up to the QC step, and restarted from there after completion of the QC analysis.

**Mapping:** Grape’s next step is the alignment of the short sequence reads to the reference genome. This step is crucial for the RNA-Seq analysis, and particular care has to be taken, as it will condition any downstream analyses. Grape alignment module is complex, performing several alignments, and then combines them into a final mapping, from which a BAM file is produced. By default, Grape uses GEM (Marco-Sola *et al.*, 2012), an exhaustive short-read mapper, allowing mismatches and indels (but other aligners could be used as long as the output format is, or can be, converted to standard BAM/SAM).

As most NGS aligners, GEM requires an ‘index creation’ step before the actual read mapping. This step preprocesses the reference sequence, creating a data structure that can be searched fast and efficiently. The choice of indices determines which reference sequences the raw reads will be searched against. The most obvious index to be used is the one corresponding to the genome sequence of the species investigated. However, when examining transcriptome data, reads mapping across splice junctions will not match the genome sequence, and it is convenient to create a specific index corresponding to the splice junctions. Thus, in addition to the genome index, Grape generates a junctions index that contains annotated splice junctions, plus all possible junctions that can be obtained by biologically legal combinations of exons within each locus. This type of approach has already been shown to distinguish different alternative splice forms when used to design splicing microarrays (Johnson *et al.*, 2003).

Grape aligns reads against both the genome and the junction references. Junction mappings are filtered to remove those reads that do not span the splice site. Next, the remaining unmapped reads are mapped using the GEM split-mapper (Marco-Sola *et al.*, 2012). This tool will attempt to divide the read into two parts and align each fragment independently to the genome. Only alignments matching consensus splice sites are further considered. This allows Grape to recover the reads mapping to unannotated ‘bona fide’ splice sites.

After this step, there may still be some unmapped reads—corresponding to unsequenced regions of the genome, genome contaminants, large number of mismatches with the references, etc. Grape follows an aggressive strategy in an attempt to reliably map as many reads as possible, and toward that end, it performs additional rounds of mapping. First, it successively increases by one (by default) the number of allowed mismatches up to a number that is proportional to the length of the read (by default, up to 1 mismatch per 25 nt of read length). For each number of mismatches, a new genome mapping (including split-mapping) is performed. Second, remaining unmapped reads are successively trimmed by a set number of nucleotides (10 by default), and a new genome (and split) mapping performed after each trimming. The aggressiveness of the trimming can be controlled by the user. A parameter sets the shortest length to which the read can be trimmed. If this length is equal to the read length, then no trimming is performed.

**Fig. 3.**
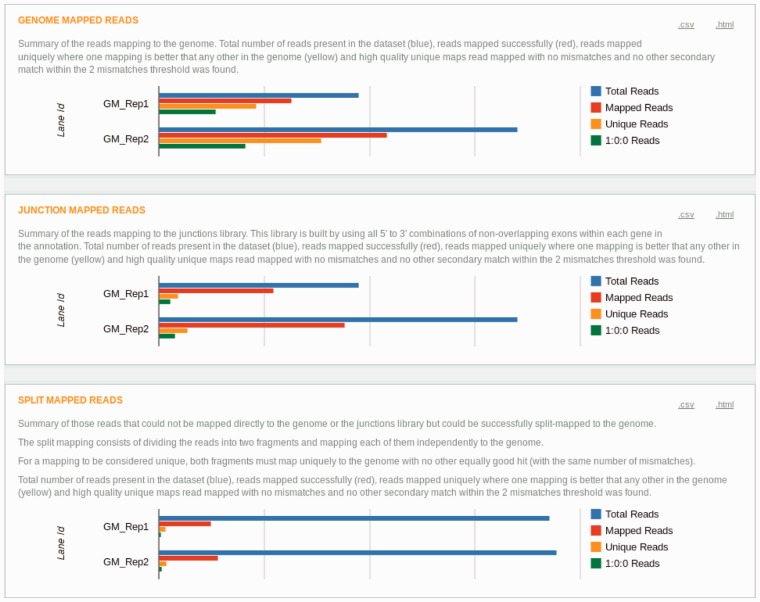
Overview of Grape’s mapping strategy. The initial genome and junctions mapping is followed by a round of split mapping. Remaining unmapped reads are aligned with additional mismatches, and the still remaining ones are iteratively trimmed. The mappings resulting from the different steps are combined into a final merged mapping

**Fig. 4.**
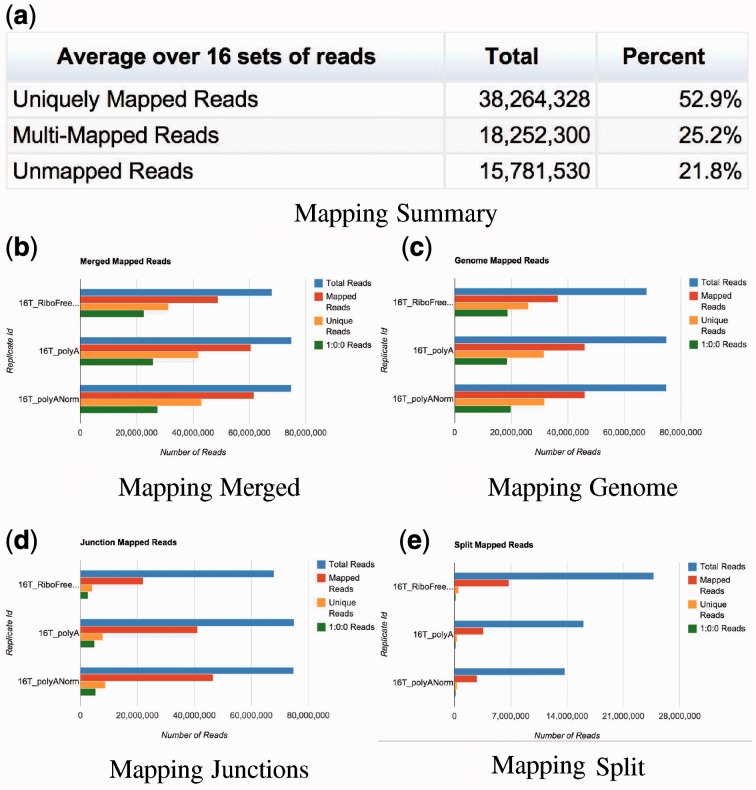
Raisin visualization of Grape’s mapping step. Panel **a** shows the overall mapping results as well as the information on the genome annotation and number of mismatches used for the alignments. Panel **b** shows the fraction of reads aligned in the final merged mapping. Panels **c**, **d** and **e** show the same type of information for the different components of the mapping process

This iterative mapping ends when all reads are mapped, or the length of the reads falls below a certain threshold (25 nt, by default). A diagram describing it can be seen in Figure 3. This approach is similar to the one described in Cloonan *et al.* (2009).The Raisin output, providing summary statistics of the Grape mappings, is shown in Figure 4.

**Post-mapping:** The reads that align in the initial round of mapping (genome, junction and split-mapping) are examined and divided into those reads that map to one location better than to any other (unique maps) and those that align to at least two positions equally well (multi-maps).

This is an important filtering step because a unique map implies higher reliability in the alignment than a multi-map. However, excluding all multi-maps from an analysis results in the potential loss of information. The eventual use of one read alignment type versus the other will depend on the type of the analysis. For example, for tasks such as the identification of novel genes, or the detection of low abundance transcripts, the confidence of the results increases if only uniquely mapped reads are considered. Other tasks, like the calculation of genome/transcriptome coverage, may use all the mapped reads.

Those reads that can be aligned to a unique position in the genome and also to a unique position in the junctions are also considered multi-maps, and are, in many cases, indicators of the presence of a pseudogene or processed copy of a gene in the genome. In the case of paired end reads, if one of the members of the pair can be aligned uniquely to a certain position, the corresponding mate can sometimes be rescued if it is a multi-map. However, in the case of RNA-Seq reads, in contrast to genomic DNA reads, the insert size cannot always be used to identify the correct mate, as the presence of introns can alter the distance between mates. Grape chooses the closest mapping position to the uniquely mapping mate in these cases. In the case of the example analyzed here, Grape assigns, on average, 52% of the reads to a unique position, whereas 25% of the reads are multi-mapping (Fig. 4a).

The results of the different mapping steps are combined into a final mapping results file in GFF, BED or SAM/BAM format. This file can be uploaded to genome browsers for visualization and comparison or be analyzed by other programs. Grape uses this file specifically for the quantification of genes and transcripts and for the delineation of transcript structures (see next).

**Transcript quantification:** Grape uses the mapping results to produce quantifications of the abundance of a number of transcribed elements: exons, splice junctions, genes and transcripts, as well as inclusion indices for exons.

**Fig. 5.**
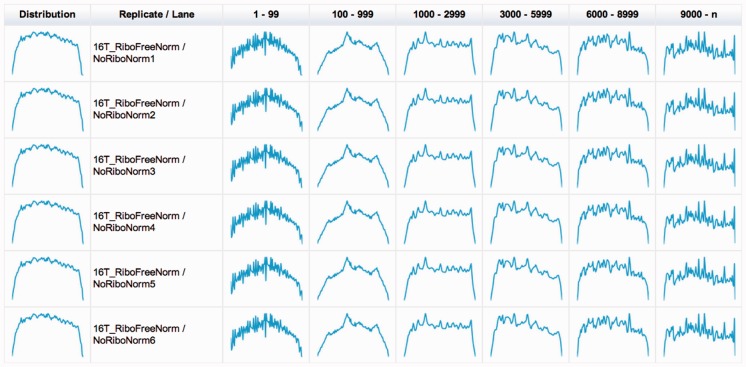
Distribution of uniquely mapping reads along the annotated transcripts. This allows us to identify biases that may be caused by issues such as RNA degradation

Before the quantification, Grape investigates the distribution of mapped reads along transcripts to identify potential 3′ to 5′ biases. Toward that end, the annotated transcripts are binned according to their length (see Fig. 5).

We use two strategies for quantifying exons and genes: (i) simple overlap, and (ii) read deconvolution. Under the first scenario, exons and genes are quantified by simply summing all the uniquely mapping reads that are fully included within the boundaries of the exon or gene. Results are given in reads per kilobase per million mapped reads (Mortazavi *et al.*, 2008). Note that reads completely included in two or more overlapping exons will be counted separately for each exon. Exon quantifications in these cases will be overestimated.

To produce quantifications of individual transcripts, we use the FluxCapacitor (Montgomery *et al.*, 2010) The FluxCapacitor converts the transcript structure of each annotated locus into a splicing graph, where junctions are represented as nodes and exons as edges. The mapping of the reads into the graph imposes a number of constraints that the FluxCapacitor represents as a system of linear equations, which can be solved using linear programming.

**Fig. 6.**
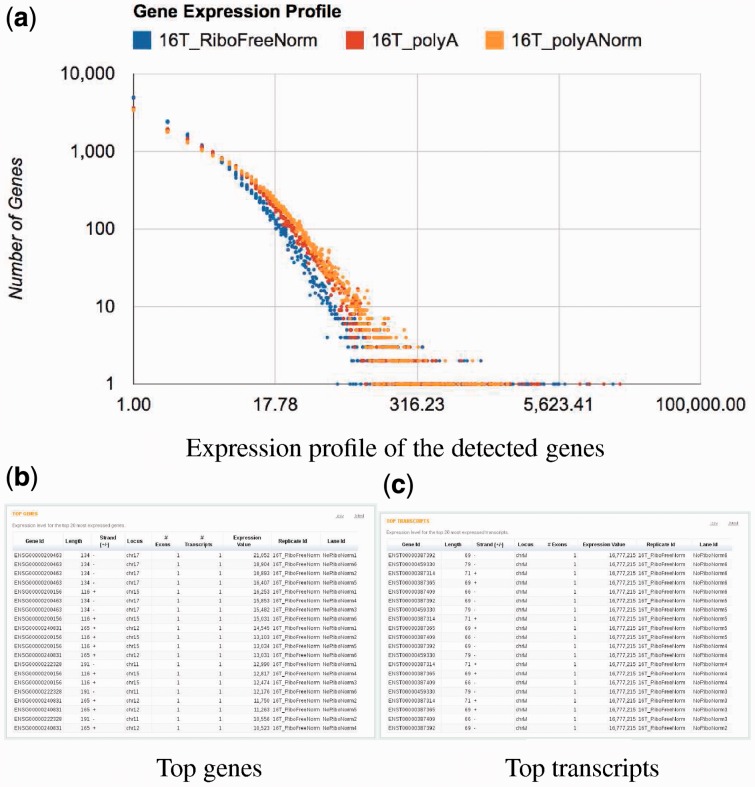
Raisin visualization of the transcript quantification step. Panel **a** shows the distribution of gene expression. Panels **b** and **c** show, respectively, the top genes and transcripts detected in the different lanes of the analyzed samples

Raisin plots the distribution of expression of all genes (Fig. 6a), and lists the top 20 highly expressed transcripts (Fig. 6c) and genes (Fig. 6b), and from the Raisin interface, it is possible to navigate to the expression values of all transcripts and genes (in html, csv and excel formats).

**Fig. 7.**
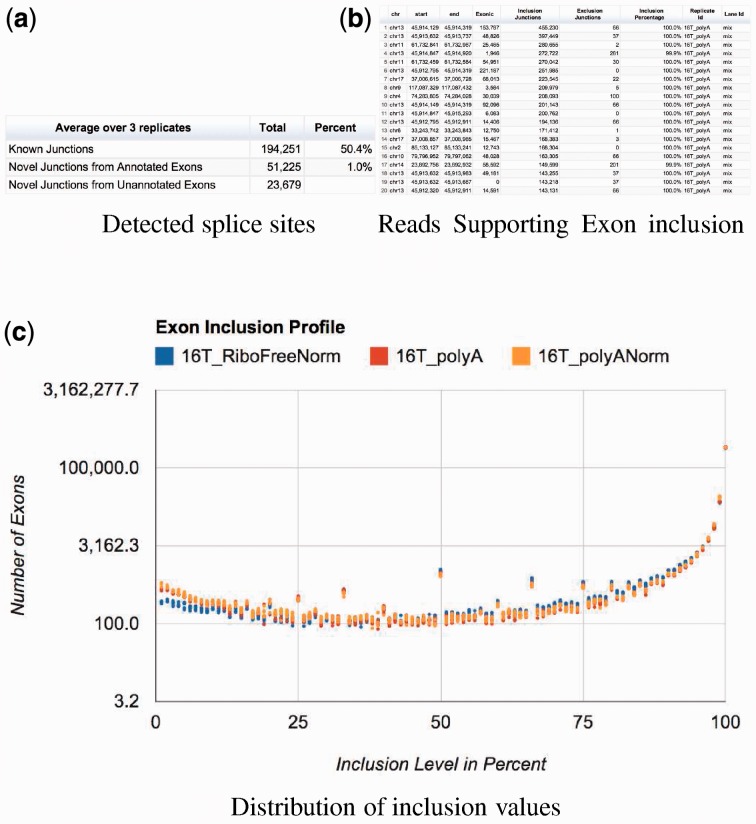
Raisin visualization of Grape’s splicing analysis. Panel **a** shows the summary table for the detected splice sites. Panel **b** contains a table with the top included exons in the samples examined, and panel **c** shows the distribution of the inclusion values over all internal exons

Splice junctions are quantified using the number of reads spanning the junction. In this case, no need for feature length normalization is required, and the choice of normalization based on number of reads in different samples is left to the criteria of the user (Fig. 7a). The detected splice sites are further classified: ‘Known’ are junctions that appear in the annotation. ‘Novel’ are junctions formed between annotated exons from the same gene, but not present in the annotation. ‘Novel from unannotated exons’ are split-map junctions, in which at least one of the two connected ‘exons’ is not annotated. In this last group, we also include any junction detected between exons from different genes.

Grape also computes an exon inclusion ratio for each annotated internal exon. Exon inclusion is computed as the ratio of all reads supporting the inclusion of the exon (reads mapping to exon junctions that include it and the exon itself) to all reads supporting its exclusion. These are reads mapping to junctions from that gene that skip the exon. See Figure 7c.

**Discovery of novel transcribed elements:** Grape runs a number of analyses to identify novel transcribed elements. First, RNA-Seq clusters are built from uniquely mapping reads, using either genomic mappings, junctions maps or split reads. There is no threshold for the number of reads that make a cluster, but Grape provides the number of reads, and of staggered reads making each cluster. Grape also detects novel splice junctions through the split mapping of reads. These are the junctions classified as novel from unannotated exons (see previously). Finally, Cufflinks (Trapnell *et al.*, 2010) is used to infer transcript structures.

Grape also implements a simple procedure to identify chimeric RNAs independently of those cases found by Cufflinks, which can be used if the input is paired end reads. Here, both mates of all read pairs mapping uniquely (with up to the specified number of mismatches) to the transcriptome are evaluated. If they map to different transcripts, they are classified as unannotated splice variants if both of these transcripts belong to the same gene. Otherwise, they map to transcripts from different annotated genes, and are classified as putative chimeric RNAs or fusions.

**Fig. 8.**
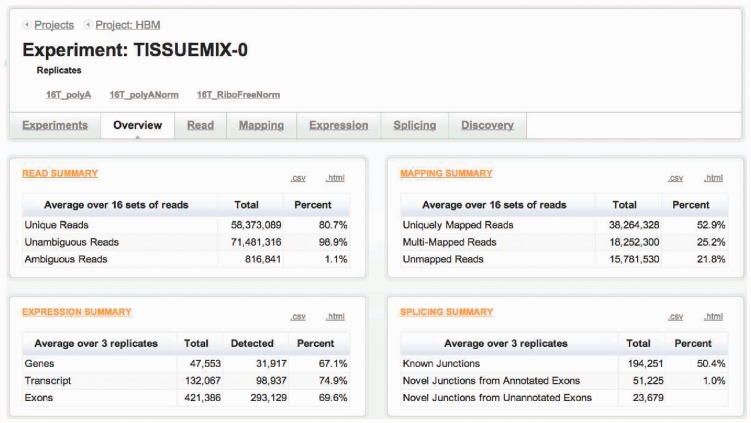
Raisin overview of Grape’s analysis results

**Summary statistics:** A page including summary statistics from all the different analysis steps in Grape is produced, and it can be accessed through Raisin (Fig. 8). From this page it is possible to navigate to each specific analysis.

**Additional analysis:** Additional analyses can be implemented within Grape by simply adding an entry to the workflow template file specifying the computations to be performed in the additional step. The new entry is a sequence of shell commands that specify the computations that need to be performed. The sequence of commands is preceded by a line that contains the name of the new step and its dependencies. These dependencies are just the names of those other steps that need to be executed before to generate the input required by the new step. For instance, in the entry

**Table:** 

preprocess: start
$BIN/preprocess.RNAseq.pl
mv preprocess.RNAseq.log $LOG

The first line contains the name of the step (‘preprocess’) and its dependencies, in this case, a step named start, and the following lines, the commands to be run. The variables set by Grape, such as *BIN and* LOG, are listed at the start of this file and can be used by any of the commands in the template file.

## 3 DISCUSSION

Here we presented and discussed Grape, an architecture for a computational pipeline for the analysis of millions or billions of short reads obtained from (potentially many) high-throughput RNA-Seq experiments. This is a general and flexible pipeline that combines contributions of previous studies with our own experience dealing with RNA-Seq data. Grape attempts to address the challenges both from the processing and management standpoints associated to the analysis of sheer amounts of data. It automates the processing and analysis steps, while at the same time providing an organizational framework that simplifies the management and summarizing of the analysis.

The full workflow of the pipeline fits in a single text file specifying the dependency graph of the pipeline’s rules. Adding a new analysis step in Grape is as simple as defining the parent steps that have to be executed beforehand, and the programs and scripts that need to be executed by the new step.

Grape’s objective is to produce quantifications of transcript abundances (and of the abundances of other transcriptional elements: genes, exons, splice junctions, etc). Grape does not perform statistical analysis and comparisons of expression or splicing usage between and/or across samples. From Grape’s output quantifications, however, other methods and tools can be used to perform such analysis [for instance, R statistical packages within Bioconductor (Gentleman *et al.* 2004; http://pypi-ranking.info/module/zc.buildou) for expression analyses, etc]. Integrating Grape’s quantification with analysis of gene expression and alternative splicing is among the further developments planned within Grape’s roadmap. In the very short-term, we plan to incorporate a method to assess data reproducibility. The method, called Irreproducible Discovery Rate (Li *et al.*, 2011), has been pioneered in the framework of the ENCODE project, and it can be applied to experiments with two replicates. Other developments include transcript assembly and quantifications in absence of a genome of reference. This could be particularly useful for species with transcriptome data, but not sequenced genome. Note that Grape can already be directly used to produce quantifications if an index from a reference transcriptome—independently assembled—is generated. Current efforts are, however, mostly focused to streamline the pipeline to maximize speed and minimize memory usage, to simplify installation of the package, to provide graphical support for interactive usage and to improve graphical reporting of the results. Currently, as of July 2012, Grape is on version 1.6, and we provide regular upgrades.

We have so far used Grape successfully for the in-house analysis of more than a thousand large RNA-Seq datasets [from projects such as ENCODE (ENCODE Consortium, 2004, 2011), Illumina Body Map (rnaseq.crg.es/project/ENCODE/tab/experiments/), GTEx (www.genome.gov/gtex), ICGC (Puente *et al.*, 2011), Geuvadis (www.geuvadis.org), Quantomics (www.quantomics.eu) and others].

Given the unique role of RNA as both a proxy and a determinant of the cellular and organism phenotype, profiling of RNA by RNA-Seq will spread beyond basic research, in medicine, agriculture, biotechnology and other technical applications of biology. RNA-Seq could be used, for instance, for continuous ambulatory monitoring of tumor response to treatment. It could become a standard component of blood tests, a single assay monitoring many more variables than current biomarker assays. Such applications of RNA-Seq require, however, analysis tools (mapping, quantification, etc) that are order of magnitude more efficient than currently existing ones. They require, in addition, robust, efficient and scalable software systems for RNA-Seq data storage, organization and analysis. The lack of such systems is often seriously limiting the utility of RNA-Seq data, and may prevent researchers to embark in medium- to large-scale RNA-Seq projects—which are otherwise within technological reach. We believe that Grape may contribute to fill such a void.
